# First-in-Human Phase II Clinical Trial of Multiplex IntraTumoral Immunotherapy (MITI) in Patients with Metastatic Solid Cancer (Abscopal 5001 Trial)

**DOI:** 10.3390/cancers17182990

**Published:** 2025-09-12

**Authors:** David G. Bostwick, Melanie M. Wilk, Brian R. Bostwick, Norman Miller, Eugene C. Rajaratnam, Junqi Qian, Peter M. Rydesky, Peter J. Littrup

**Affiliations:** 1 Rampart Health, 4724 Lake Calabay Drive, Orlando, FL 32837, USA; mwilk@ramparthealth.com (M.M.W.); normmiller21@gmail.com (N.M.); junqiqian@yahoo.com (J.Q.); pjlittrup@gmail.com (P.J.L.); 2 Antelope Valley Surgical Institute, Lancaster, CA 93534, USA; gene.raj@gmail.com; 3 Ascension Providence Rochester Hospital, Rochester, MI 48307, USA; peter.rydesky@ascension.org

**Keywords:** metastases, cancer, intratumoral, immunotherapy, clinical trial, phase II, abscopal, PD-1 inhibitor, checkpoint inhibitor, prospective

## Abstract

**Background/Objectives:** Little is known about the synergy between intratumoral immunotherapy and cancer ablation. We conducted a Phase II Trial (Abscopal 5001 trial; NCT04713371) in patients with metastatic solid cancer to assess the safety and efficacy of cryoablation with concurrent injection of RPT-01-5001 (combination of low-dose checkpoint inhibitors and cyclophosphamide), a treatment process referred to as Multiplex Intratumoral Immunotherapy (MITI^TM^). **Methods:** Twelve patients with metastatic cancer who had failed standard therapy and one with sacral chordoma received at least one intratumoral treatment of MITI preceded by 3–5 days of oral low-dose cyclophosphamide. MITI consisted of CT-guided cryoablation followed by intratumoral injection of RPT-01-5001. GM-CSF was subcutaneously administered daily for four weeks. Treatment was repeated every four weeks if the tumor burden remained stable or reduced, as noted by the iRECIST criteria. These criteria were modified when follow-up biopsies revealed pathology with minimal or no cancer, despite persistent suspicious masses on imaging. **Results:** Cancers included prostate (four patients), sarcoma (two), and one each of breast, colon, bladder, uterine cervix, tongue, kidney, and sacral chordoma. Eight patients received three cycles of treatment, two received two, and three received one. All patients tolerated the procedure well and were discharged within 2 h. The adverse event rate was 69%, all of which were grade 1 or 2, except for two grade 3 cases with delayed cryosurgical complications (15%). At completion of up to three cycles of treatment, a complete response (iCR) was observed in one patient (7.7%), partial response (iPR) in four patients (30.8%), and stable disease (iSD) in five (38.5%), with a disease control rate (iDCR) of 77%. Disparity between post-treatment imaging and pathologic findings was observed in four patients (positive vs. negative, respectively), requiring modification of the iRECIST criteria in favor of pathology. The best response ranged from 0 to 91%, with a mean for responding patients of 38%. Median progression-free survival (PFS) and 95% confidence intervals (95% CI) were 5.4 months (1.8 to 23.1 months); and median overall survival (OS) was 20.9 months (9.1 to 22.8 months). The injection site cancer response was observed in nine (69%) patients, and the distal abscopal effect was seen in four (31%), including one sarcoma patient with a complete abscopal response of lung metastases and one bladder cancer patient with biopsy-confirmed complete resolution of lung and liver metastases. **Conclusions:** MITI with RPT-01-5001 is safe and highly feasible, providing 77% disease control and 31% of the abscopal effect in patients with metastatic cancer who have failed standard therapies.

## Simple Summary

Immunotherapy with multiple checkpoint inhibitors has not been previously studied when administered intratumorally in combination with cancer ablation. This approach was used to simultaneously exploit four complementary mechanisms of anti-tumoral immunostimulation in order to improve the treatment response in a range of metastatic cancers. The aim of our prospective phase II clinical trial was to assess the safety and efficacy of this multiplex salvage therapy in a variety of metastatic cancers that have failed standard therapies. We confirmed, in a group of 13 patients, that this approach is safe and highly feasible, providing 77% disease control and 31% abscopal effect. The adverse event rate ≥ 3 was seen in 15% of cases. Intraumoral immunotherapy, in combination with other forms of anti-cancer immunostimulation such as ablation, shows promise as an alternative to systemic single-agent immunotherapy.

## 1. Introduction

Metastatic cancer is an enormous health problem, and first- and second-line therapies, such as chemotherapy and immunotherapy, are rarely curative. Thus, there remains a great unmet medical need for an effective and durable treatment.

We developed a novel Multiplex IntraTumoral Immunotherapy (MITI™) approach to improve the treatment response in a range of metastatic cancers. MITI is a unique, multi-pronged, mechanistically-driven therapy that offers multiple advantages unavailable with systemic therapy, including the creation of a personalized in situ antigen source and immunization to generate and augment antitumor immunity in close proximity to injected medications while reducing the risk of widespread toxicity [1,2]. Unlike systemic immunotherapy, MITI treatment is localized to cancer, avoiding most immunotherapy-related adverse events. The RP-01-5001 combination of drugs (pembrolizumab, ipilimumab, and cyclophosphamide) is thought to generate a significant CD8+ memory antitumor immune response, triggering a systemic CD4 and CD8 T cell-mediated response capable of eradicating distant metastases (abscopal effect) [3].

Compared with other intratumoral immunotherapy modalities, MITI represents a fundamentally distinct approach that extends beyond simply combining agents. Most prior intratumoral strategies have relied on a single-drug checkpoint blockade, viral vectors, oncolytic viruses, or cytokine injections, each limited by narrow mechanistic focus, unpredictable antigen release, or systemic toxicity when escalated. Radiation- or ablation-assisted protocols have shown some synergy with intratumoral drugs but typically exploit only one or two immune-activating mechanisms. In contrast, MITI with RP-01-5001 deliberately integrates five complementary immunostimulatory processes in a coordinated, mechanistically rational sequence (Table 1): controlled cellular lysis (cryoablation) to release the full payload of intact, personalized antigens, damage-associated molecular patterns (DAMPs), cytokines, and other immunostimulants; intratumoral PD-1 blockade to reverse exhaustion and expose cancer-specific antigens to dendritic cells and cytotoxic (killer) T-cells while facilitating T-cell antitumor activity; intratumoral CTLA-4 blockade and low-dose cyclophosphamide to suppress inhibitory signals from regulatory T-cells and potentiate and prolong the antitumor cytotoxic T-cell response; subcutaneous GM-CSF to mobilize and sustain effector T cells, as well as suppress inhibitory signals from T-regulatory cells; and the multiplex combination itself to amplify systemic immune activation and reliably induce abscopal effects. This architecture is novel not only because of the multiplexity but because it purposefully preserves vascular and stromal integrity at the treatment site to maximize antigen presentation and trafficking and incorporates a histopathology-driven refinement of response assessment to address the challenge of pseudoresiduum—a limitation overlooked in most intratumoral trials. Thus, MITI establishes a new paradigm in intratumoral immunotherapy by creating an in situ, patient-specific vaccine while minimizing systemic toxicity and by demonstrating both feasibility and meaningful abscopal responses across diverse metastatic cancers.

This research builds on a previous proof-of-concept study showing that MITI can improve outcomes in patients with metastatic cancer who have failed standard therapies [1]. In a retrospective report of 19 patients treated with MITI for metastatic prostate cancer, the complete response was 47% and the disease control rate was 73%. Responses were also observed in bladder cancer (two patients), melanoma (one patient), and colon cancer (one patient).

In this first in-human phase 2 clinical trial, we assessed the safety and efficacy of MITI in a population of patients with metastatic cancer as salvage therapy for those who have failed conventional treatments. Our results indicate that Multiplex Intratumoral Immunotherapy (MITI) with RPT-01-5001 is a safe and highly feasible outpatient treatment for multiple cancer types with a favorable adverse event profile. The abscopal effect is a common and welcome finding.

## 2. Methods

Prior to initiation, the study was cleared by the U.S. Food and Drug Administration, registered with the National Library of Medicine (clinicaltrials.gov, NCT04713371), and approved by the Institutional Review Board, Advarra, Inc. (Columbia, MD, USA).

### 2.1. Patients

All patients were enrolled and treated at a single institution. The study population consisted of adults (≥18 years) with metastatic solid cancer with at least one imaging or histologically-proven metastasis to lymph nodes, bone, or soft tissue measuring at least 1.0 cm in diameter. All patients had failed conventional therapy; none had received previous treatment that specifically targeted T-cell co-stimulation or checkpoint pathways, although this was not an exclusion criterion for entry. Most were eligible for hospice care, but none participated at the time of entry. Life expectancy of more than 3 months was required, as well as a performance status of 0–3 on the ECOG Performance Scale. Participants were obliged to abstain from prior use of other immunotherapeutic or chemotherapeutic agents or treatments for at least two weeks and throughout the course of treatment. The exclusion criteria included hypersensitivity to any of the medications in the protocol, concurrent medications for cancer within 4 weeks of initiation, including steroids, active infection or autoimmune disease, pregnancy, or additional malignancy that is progressing or requires active treatment; however, none of these exclusion conditions were encountered during screening.

Patients and the public were not involved in any way in the design or conduct of the study, choice of outcome measures, recruitment, or dissemination of the study results.

### 2.2. Clinical Trial Design

The Abscopal 5001 trial was a first-in-human, single-arm, non-blinded phase II study of Multiplex Intratumoral Immunotherapy (MITI™) in patients with metastatic solid cancers refractory to standard therapies. The primary endpoint was progression-free survival, with secondary endpoints including disease control rate, overall survival, abscopal response, and adverse event profile.

All treatments were performed at Ascension Providence Rochester Hospital, Rochester Hills, MI, USA. The radiologic images of potential patients were reviewed by board-certified interventional radiologists (PL and PR) to determine whether a safe CT-guided route existed for combined cryoablation and infusion. For each cycle, low-dose oral cyclophosphamide (50 mg) was administered daily for 3–5 days immediately before MITI treatment. MITI was initiated by placing a 1.7 mm diameter short freeze-segment probe (IceSeed; Galil Medical Inc., St. Paul, MN, USA) using CT guidance into the targeted tumor(s) in up to two cancer sites to create ~2.0 cm diameter iceballs using a single cycle of ~2–3 min of cryosurgical freezing, thereby inducing intratumoral controlled cellular lysis. No effort was made to ablate the entire cancer mass. While waiting for the freeze to develop, a 17-gauge trocar needle was placed parallel to the cryoprobe to reside approximately 10 mm from the cryoprobe. After a two-minute delay to allow thawing, the cryoprobe was removed, and each ablated site was slowly injected with three medications comprising RP-01-5001: 50 mg (2 cc) of the PD-1 inhibitor monoclonal antibody pembrolizumab (Keytruda, Merck & Co., Inc., Rahway, NJ, USA); 10 mg (2 cc) of CTLA-4 inhibitor antibody ipilimumab (Yervoy, Bristol Myers-Squibb, NY, USA); and 20 mg (1 cc) of cyclophosphamide (Baxter Healthcare, Largo, FL, USA). Subsequently, 4 cc of collagen hydrogel (Helitene, Integra LifeSciences Corporation, Plainsboro, NJ, USA) was injected at the treatment sites to mitigate back-oozing of the solutions and aid in hemostasis. Sargromostim (GM-CSF; 250 mcg^2^/day) (Leukine, Partner Therapeutics, Waltham, MA, USA) was subcutaneously administered daily for 4 weeks. Each patient received up to 3 treatment cycles at 4-week intervals.

Patients were monitored through scheduled clinic visits and laboratory studies before each cycle as clinically indicated. Imaging was performed during each treatment by CT scan and intermittently after treatment. Other imaging modalities (e.g., ultrasound, MRI, and PSMA scans in patients with prostate cancer) were performed at the discretion of the treating physician. Biopsies were performed at the discretion of the treating physician, usually at the onset of treatments 2 and 3. Blood tests included CBC with differential, comprehensive metabolic blood panel, and liver function tests. Patients remained on treatment until disease progression or the treatment protocol of three cycles was completed.

### 2.3. Toxicity Assessment

The incidence of adverse events was assessed using the National Cancer Institute Common Terminology Criteria for Adverse Events (NCI-CTCAE), version 5.0. Adverse events were monitored beginning on day 1 of cycle 1 and continuing until 30 days after the last cycle, with cumulative toxicity reported. Patients were required to maintain a daily calendar that included details of sargromostim (GM-CSF) self-administration and any adverse events.

### 2.4. Clinical Response Assessment

The response was assessed using the modified RECIST1.1 and iRECIST criteria [4]. In cases in which there was disparity between CT imaging and histopathology, preference was given to histopathology findings to avoid false–positive imaging errors.

### 2.5. Statistics

This trial was conducted to determine the feasibility, safety, and efficacy of MITI in a variety of solid cancers. Progression-free survival (PFS) and overall survival (OS) were evaluated using Kaplan–Meier tests.

## 3. Results

Twelve patients with metastatic cancer and one patient with sacral chordoma were enrolled in the study (Table 2). The mean age was 67.2 years (range, 38–87 years). Four patients were female (30.8%). Cancers included prostate (four patients), sarcoma (two), and one each of breast, colon, bladder, uterine cervix, tongue, kidney, and sacral chordoma (Table 2). Eight patients received three cycles of treatment, two received two, and three received one. All patients tolerated the outpatient procedure well and were discharged within 2 h.

### 3.1. Adverse Events

The adverse event rate was 69%, and all were grade 1 or 2, except for two transient grade 3 (15%) cryosurgical complications (transient pneumothorax and transient pneumonia immediately following MITI that did not require hospitalization) (Table 3). Skin rash and transient diarrhea were the most common events attributed invariably to the administration of sargromostim (GM-CSF).

### 3.2. Outcomes

**Figure 1:**
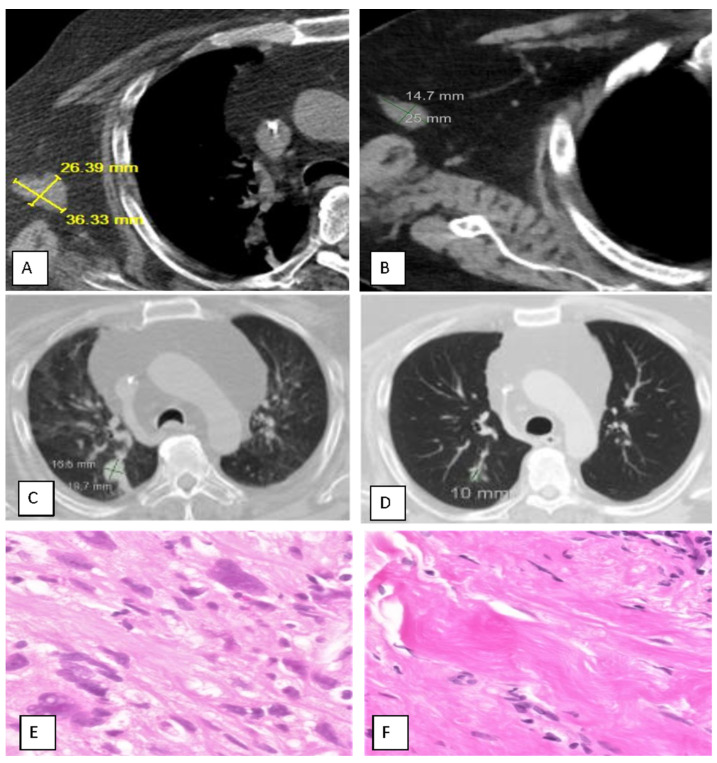
Case Study of a Patient Successfully Treated with MITI for Metastatic Fibrosarcoma. (**A**,**C**,**E**), Before treatment; (**B**,**D**,**F**), After treatment. CT SCAN: Left arm stump mass. (**A**): 36.3 × 26.4 mm = 958.2 mm^2^ (abnormal; all cancer); (**B**): 25 × 14.7 mm = 367 mm^2^ (substantial shrinkage; complete resolution of cancer according to biopsy (see (**F**), below)). CT SCAN: Lung, Right Lower Lobe mass. (**C**): 19.7 × 15.5 mm = 305.4 mm^2^ (abnormal); (**D**): 10 × 8 mm = 80 mm^2^ (normal) (substantial shrinkage; complete resolution of cancer); Biopsy Histopathology: Left Arm Stump. (**E**): Cancer: Myxoid Fibrosarcoma; (**F**): Benign Fibrous Pseudotumor.

Upon completion of up to three cycles of treatment, complete response (iCR) was observed in 1 patient (7.7%), partial response (iPR) in 4 patients (30.8%), and stable disease (iSD) in 5 (38.5%), for a disease control rate (iDCR) of 77%; progression was observed in 23% (Table 2). The best response ranged from 0 to 91%, with a mean for responding patients of 38%. The injection site response was observed in nine (69%) patients, and a distal abscopal effect was seen in four (31%), including one sarcoma patient with a complete abscopal response of lung metastases (Figure 1) and one bladder cancer patient with resolution of lung and liver metastases.

Seven patients underwent biopsy of CT-positive presumptive residual cancer at sites of prior MITI treatment, but only three were positive. The other four (57%) had shrunken residual masses of fibrous pseudotumor, referred to as pseudoresiduum (defined as a shrunken mass of fibrous tissue that has replaced the prior cancer; “pseudo” signifies the false–positive interpretation by radiologic study); all four had partial or complete response. This disparity between post-treatment imaging and pathologic findings required modification of the iRECIST criteria, with reliance placed on histopathology when available.

**Figure 2:**
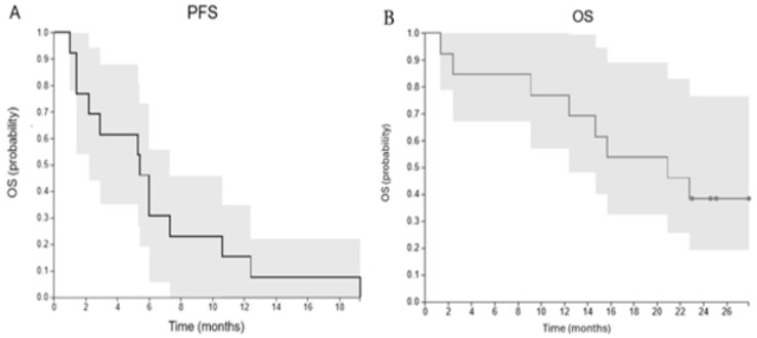
Survival Analysis. (**Left**): Progression-free survival. Median PFS = 5.4 (1.8 to 23.1) months; (**Right**): Overall Survival. Median OS = 20.9 (9.1–22.8) months.

Median progression-free survival (PFS) and 95% confidence intervals (95% CI) were 5.4 months (1.8 to 23.1 months) (Figure 2), although PFS is a challenging endpoint in the context of immunotherapy due to confounding pseudoresiduum or pseudoprogression. The median OS and 95% CI were 20.9 months (9.1 to 22.8 months) (Figure 2).

An adult patient with a long history of myxoid fibrosarcoma (grade 4 of 4) required left arm above-the-elbow amputation and radiotherapy to the left rib in 2020; however, he was intolerant to chemotherapy. By 2021, he had widespread metastases, including right lung nodules, right axillary lymph nodes, soft tissues of the left arm (stump), and a recurrent left rib. He underwent two cycles of MITI, resulting in the resolution of all but rib metastases within 2 months. The only adverse event was transient injection site discomfort, which was treated with acetaminophen.

## 4. Discussion

This small prospective study must be interpreted with caution; however, several important conclusions can be drawn regarding feasibility, safety, and efficacy. First, Multiplex IntraTumoral Immunotherapy (MITI) with RP-01-5001 is highly feasible in patients with metastatic cancer who have failed standard therapies. Second, the intratumoral route of administration is safe and well tolerated in patients with various types of cancer, with a favorable adverse event profile. In addition to skin rash and diarrhea, this approach obviates typical immune-related events of systemic immunotherapy, such as endocrinopathies and liver toxicity. Finally, we found MITI with RP-01-5001 to be efficacious, with a disease control rate of 77%, abscopal effect of 31%, and median overall survival of 20.9 months.

These findings confirmed our previous anecdotal findings with metastatic prostate cancer [1]. In an observational retrospective study of 18 patients with metastatic prostate cancer (NCT03695835), MITI yielded an unprecedented disease control rate of 73%, with 47% of patients achieving a complete response [1]. Additionally, the incidence of serious adverse events (grade 3–4) was 19%, indicating the safety of MITI Therapy. That report used a variation in RP-01-5001, in which sargramostim (GM-CSF) was used intratumorally (as well as subcutaneously) in place of cyclophosphamide.

MITI with RP-01-5001 is based on five distinct and complementary immunostimulatory mechanisms to optimize and personalize the antitumor effect (Table 1). First, controlled cellular lysis (cryoablation) releases all cancer antigens—not just one or a few—that are present throughout the patient’s cancer by carefully controlling the induction of necrosis and necroptosis, which limits the extent of cell and membrane destruction while liberating individualized cancer-specific antigens to maintain their structural integrity and maximum antigenicity. Local destruction is reduced to a ≤ 1.0 cm diameter focus within each treated cancer site, thereby preserving the blood/lymphatic vasculature to allow entry and exit of the full repertoire of immune cells and ensure intimate contact of the immune system with both damaged and undamaged cancer cells, damage-associated molecular patterns (DAMPS), cytokines, and other immunostimulants released from cancer cells. This discharge of antigens activates a strong, systemic, and tumor-specific immune response. After the entire payload of tumor-associated antigens is released, immature dendritic cells are activated and begin the process of recruiting cytotoxic T cells that surround and destroy cancer cells. The second mechanism of immunostimulation, “unmasking” of cancer antigens by the introduction of PD-1 inhibitors, exposes these foreign antigens to dendritic cells and cytotoxic (killer) T-cells while facilitating T-cell antitumor activity; it also reverses T-cell exhaustion. This, in turn, triggers a systemic response that results in the destruction of cancer cells elsewhere in the body that express the same antigens (the abscopal effect). The third mechanism involves the delivery of anti-CTLA-4 and low-dose cyclophosphamide to suppress inhibitory signals from regulatory T-cells and potentiate and prolong the antitumor cytotoxic T cell response. Collagen hydrogel is also injected to minimize the leakage of drugs back along the needle tracts and/or into the interstitium. The fourth mechanism of immunostimulation is induced by daily metronomic injection of sargromostim (GM-CSF) to mobilize immune cells, especially cytotoxic T-cells, from the bone marrow, suppress inhibitory signals from T-regulatory cells, and prolong the antitumor cytotoxic T-cell response. GM-CSF has a variety of other effects on the immune system, including the activation of T cells, maturation of dendritic cells, and promotion of humoral and cell-mediated responses. The final mechanism of immunostimulation involves harnessing the power of the abscopal effect. Our initial proof-of-concept studies revealed a high incidence of the abscopal effect, likely as a result of the activation of immune cells within the cancer stroma that then drain to regional lymph nodes, triggering a systemic response that culminates in the destruction of cancer cells elsewhere in the body [1]. The abscopal effect is a long-distance (up to tens of centimeters outside the treatment field) systemic effect at a distant metastatic site that is mediated through immunogenic responses. Recent studies have shown an increase in the incidence of the abscopal effect after cancer irradiation following the administration of immunotherapy [5,6,7]. Golden et al. were the first to report the synergy of radiation therapy, systemic immunotherapy, and chemotherapy, inducing an abscopal effect in 27% of patients with metastatic solid cancer and extending survival (21 vs. 8 months) [8]. In the current report, we found a remarkably similar incidence of the abscopal response (31%) and overall survival (20.9 months).

The intratumoral route of drug administration was chosen to allow the close proximity of tumor antigens to the immune process and to elicit fewer side effects than systemic therapy. MITI with RP-01-5001 resulted in an adverse event rate of 69%, all of which were grade 1 or 2, except for two transient Grade 3 (15%) cryosurgical complications (Table 1). Skin rash and diarrhea were the most common events invariably attributed to the administration of GM-CSF. This compares favorably with the historical incidence of adverse events due to treatment (trAE) and immune responses (irAE) in cancer patients receiving second-line or salvage therapy, such as chemotherapy and/or systemic immunotherapy. For example, in patients with metastatic bladder cancer who failed primary therapy and received second-line systemic PD-1/L1 therapy, the pooled incidence rates were 70% for any-grade trAEs, 17% for grade 3 trAEs, 25% for any-grade irAEs, and 8% for grade 3 irAEs [9]. Also, second-line chemotherapy and systemic immunotherapy in metastatic cancers, such as bladder cancer, only extend life minimally, and the quality of life for the extra months is suboptimal. Salvage therapy has an even poorer outcome profile.

Radiographic imaging after systemic immunotherapy often underestimates the objective response compared with the postoperative pathological response [10,11] and there is a similar high level of false–positive errors after intratumoral immunotherapy. Seven of our patients underwent biopsy of CT-positive presumptive residual cancer at sites of prior MITI treatment, and four (57%) had shrunken residual masses of fibrous pseudotumors, all with a partial or complete clinical response. The term pseudoresiduum is defined as a contracted mass of fibrous tissue that radiographically mimics cancer, as seen here; in contrast, pseudoprogression (which was not observed in this study) refers to an enlarged mass. Recognizing that pseudorersiduum is a common finding after MITI, we recommend that a biopsy be required and that iRECIST criteria be modified with reliance placed on histopathology rather than imaging. False–positive misinterpretation increases the risk of unnecessary repeat surgery and other therapies and should be avoided.

Our study is subject to several limitations that warrant consideration. First, the patient cohort was both small and heterogeneous, and the absence of a control arm restricts the robustness of efficacy assessments, necessitating cautious interpretation of the findings. Second, the rate of treatment discontinuation was relatively high (38%; 5 of 13), with most patients withdrawing after only a single dose. Notably, none of these early withdrawals underwent post-treatment tumor biopsies, raising the possibility that some of the observed radiographic responses may represent false–positive findings. Third, nearly all participants traveled from out of state to receive MITI therapy, and it is uncertain whether this geographic factor contributed to diminished retention and follow-up participation. In addition, a number of patients received subsequent conventional therapies following disease progression, which likely confounded the interpretation of overall survival outcomes. Furthermore, the intratumoral drug doses employed in this trial were empirically determined from phase 1 experience and largely extrapolated from published data on intravenous and parenteral administration and thus may not reflect the true optimal dosing strategy for intratumoral delivery. Finally, the sample sizes of individual subgroups were too small to allow meaningful statistical analysis, as any results would have been unreliable and potentially misleading; therefore, subgroup analyses were not conducted. Collectively, these limitations underscore the need for larger, controlled studies that can refine dosing, reduce attrition, and more definitively establish the therapeutic potential of MITI as a novel treatment approach.

## 5. Conclusions

This first-in-human prospective clinical trial of Multiplex Intratumoral Immunotherapy aimed to expand our understanding of a novel method of salvage treatment that avoids many of the toxicities associated with traditional systemic cancer immunotherapy and simultaneously exploits multiple complementary mechanisms of stimulation of the immune system, leading to a localized tumor-targeted personalized immune response and, frequently, an abscopal response. We propose that MITI with RP-01-5001, a fundamentally new mechanistically based approach, is feasible, safe, and efficacious in many patients requiring salvage therapy and is often able to harness the abscopal effect.

## Figures and Tables

**Table 1:** Multiplex Intratumoral Immunotherapy (MITI) with RP-01-5001: Agents and Mechanisms of Action.

Agent	Mechanism of Action	Description
Cryoablation	Releases Patient-specific Cancer Neoantigens	Damages and kills cancer cells, releasing cancer neoantigens with partially or fully intact protein structures. Cytokines and DAMPs (damage-associated molecular patterns) are also released into the extracellular matrix.
Pembrolizumab (PD-1 inhibitor) *	Unmasks Cancer	Blocks the interaction between PD-1 and PD-L1, two proteins that suppress the immune system, thereby allowing cytotoxic T cells to kill cancer cells.
Ipilimumab (CTLA-4 inhibitor) and low-dose Cyclophosphamide *	Releases Brakes on Immune System	Blocks the interaction between the CTLA-4 protein on T cells and its ligands (CD80 and CD86) on antigen-presenting cells, essentially preventing CTLA-4 from suppressing T cell activation, thereby allowing the immune system to more effectively attack cancer cells by releasing the “brakes” on the T cell response. Low-dose cyclophosphamide promotes antitumor immunity by selectively depleting regulatory T cells and enhancing effector T cell function.
Low-dose Cyclophosphamide and GM-CSF	Stimulates Immune System	Low-dose cyclophosphamide promotes antitumor immunity by selectively depleting regulatory T cells and enhancing effector T cell function. GM-CSF promotes cell survival through phosphatidylinositol-3-kinase (PI3K) and JAK/STAT5-Bcl-2 signaling and induces cell proliferation mainly by Erk and NF-kB signaling.
Combination of all treatments	Harnesses the Power of the Abscopal Effect	Precise biological mechanism is unknown but it is likely that the immune system is integral in the abscopal response.

* The combination of intratumoral drugs (pembrolizumab, ipilimumab, and cyclophosphamide) constitute RP-01-5001.

**Table 2:** Patient Data: Abscopal 5001 Trial.

Patient No.	Diagnosis	Sites of Involvement	# MITI Treatments	Adverse Events	iRECIST Status at 1 Month	PFS (Mos.)	OS (Mos.)
1	Myxoid Fibrosarcoma	Right shoulder; Right pelvic lymph nodes; Left calf; Liver; Adrenal; Lungs; Left forearm	1	None	Progression; No follow-up imaging; Withdrawal owing to comorbidities	1.3	23.0 (alive)
2	Prostate cancer (Gleason 4 + 5 = 9 adenocarcinoma)	Prostate; pelvic lymph nodes; bone and spine	1	Grade 1: Transient injection site skin rash	Withdrawal owing to co-morbidities; Modest shrinkage of treated lymph nodes	1.4	2.4
3	Colon cancer (Grade 3 adenocarcinoma)	Lungs, liver, lymph nodes	1	Grade 3: Transient procedure-induced pneumothorax; mild chest wall pain	Withdrawal owing to radiologic progression	1.8	3.6
4	Kidney cancer (Grade 2 clear cell renal cell carcinoma)	Lymph nodes (supraclavicular, mediastinal, pelvic); lungs	2	Grade 3: Injection site pain; bronchitis; gastric discomfort; diarrhea; transient pneumonia	Stable (7% shrinkage of cancer)	6.3	9.1
5	Myxoid Fibrosarcoma (Grade 3)	Stump, axillary lymph nodes, lungs, rib	2	Grade 1: Transient injection site skin rash	Partial Response; Abscopal effect in lungs (complete) and rib (partial)	10.6	20.9
6	Cervical Cancer (Grade 3 squamous cell carcinoma)	Cervix, vaginal cuff, lymph nodes	3	Grade 2: Pelvic pain; transient injection site pain; transient right leg numbness	Partial Response (near-complete); Abscopal effect in lymph nodes and vaginal cuff	7.3	22.8
7	Sacral chordoma	Contiguous spread to base of spine, perianal region, and thigh	3	Grade 2: Injection site skin rash	Partial Response; Abscopal effect in multiple nodules in thigh	19.3	27.9 (alive)
8	Bladder Cancer (Grade 3 urothelial carcinoma)	Bladder, bilateral lungs, liver, para-aortic lymph nodes, spine	3	None	Complete response; Abscopal effect in bladder, lungs and liver; Complete resolution of liver and lymph node metastases; Markedly better breathing one week following the first MITI session that was durable, likely due owing to resolving obstruction and atelectasis as the cancer shrank.	5.4	15.7
9	Oral cancer (Grade 3 squamous cell carcinoma)	Tongue cervical lymph nodes	3	Grade 1: Skin rash at injection site, dry mouth; Herpes recurrence	Stable; 14% decrease in lymph node volume; no new lesions	12.4	24.6 (alive)
10	Breast cancer (Grade 2 ductal carcinoma)	Left adrenal; liver; pelvic lymph nodes; spine and bones	3	Grade 2: Hematoma; Back pain; nausea; diarrhea; constipation	Stable; no new lesions, no increases in size	5.3	12.4
11	Prostate cancer (Gleason 3 + 4 = 7 adenocarcinoma)	Prostate, seminal vesicles, ischiorectal soft tissues, liver, bones	3	None	Stable; no new lesions	6.1	23.0 (alive)
12	Prostate cancer (Gleason 4 + 3 = 7 adenocarcinoma)	Prostate; pelvic lymph nodes	3	None	Partial Response; (44% decrease in lymph node volume (PSA dropped from 20.7 ng/mL to 13)	2.2	14.7 (alive)
13	Prostate cancer (Gleason 4 + 5 = 9 adenocarcinoma)	Prostate; pelvic lymph nodes	3	Grade 1: Diarrhea; transient hematochezia	Stable; Serum PSA dropped from 80 to 36 ng/mL; no new lesions	2.9	25.1 (alive)

**Table 3:** Adverse events after MITI in Abscopal 5001 Trial of Metastatic Solid Cancer.

Adverse Events (AEs)	Metastatic Solid Cancers (n = 13)
Grade 1–2 AEs	54% (7/13)
Skin rash	23% (3/13)
Diarrhea	23% (3/13)
Injection site pain	8% (1/13)
Urinary tract infection	8% (1/13)
Nausea	8% (1/13)
Constipation	8% (1/13)
Hematoma at injection site	8% (1/13)
Recurrent Herpes outbreak	8% (1/13)
Bronchitis	8% (1/13)
Back pain	8% (1/13)
Transient right leg numbness	8% (1/13)
Grade 3 AEs	15% (2/13)
Transient pneumothorax	15% (2/13)
Any grade	69% (9/13)
Deaths related to therapy	0

## Data Availability

The data are available upon request. All data relevant to the study were included in the article.
